# Immediate Effects of Whole-Body versus Local Dynamic Electrostimulation of the Abdominal Muscles in Healthy People Assessed by Ultrasound: A Randomized Controlled Trial

**DOI:** 10.3390/biology12030454

**Published:** 2023-03-16

**Authors:** Lorena Álvarez-Barrio, Vicente Rodríguez-Pérez, César Calvo-Lobo, Raquel Leirós-Rodríguez, Eduardo Alba-Pérez, Ana Felicitas López-Rodríguez

**Affiliations:** 1 Department of Nursing and Physiotherapy, Universidad de León, 24400 Ponferrada, Spain; 2 Department of Nursing and Physiotherapy, Universidad de Salamanca, 37008 Salamanca, Spain; 3 Faculty of Nursing, Physiotherapy and Podiatry, Universidad Complutense de Madrid, 28040 Madrid, Spain; 4 SALBIS Research Group, Department of Nursing and Physiotherapy, Universidad de León, 24400 Ponferrada, Spain

**Keywords:** electrostimulation, EMS, whole body electrostimulation, WB-EMS, abdominal muscle, ultrasonography, RUSI, muscle thickness, heart rate, active straight leg raise test

## Abstract

**Simple Summary:**

The muscles of the abdominal wall play a fundamental role in the stabilization of the pelvis and the spinal column, and they must function properly. The simultaneous combination of physical exercise with electrical currents, called dynamic electrostimulation, can have beneficial effects on this musculature in terms of gaining muscle mass and strength. Our research aimed to determine the immediate effects of a single session of dynamic electrostimulation on the thickness of the abdominal musculature and the inter-rectus distance evaluated by ultrasound, as well as on the physiological parameters of heart rate, blood pressure, and body temperature. In addition to the possible differences according to the way of application—local with electrodes placed in the abdominal area or global with whole-body electrostimulation—a total of 120 healthy participants were randomly divided into three groups: WB-EMS, EMS, and control groups. No differences were found in the results of the variables analyzed between the groups, except for heart rate. The EMS group showed a smaller increase in post-intervention heart rate compared to the WB-EMS and control groups. The use of localized dynamic EMS on the abdominal musculature in populations with cardiorespiratory disorders could be of interest, and more research is needed.

**Abstract:**

Dynamic electrostimulation consists of the application of local or global electrostimulation together with physical exercise. This study aimed to investigate the immediate effects of a dynamic electrostimulation session on the thickness of the abdominal musculature, inter-rectus distance, heart rate, blood pressure, and body temperature, and to identify possible differences in its form of application. A total of 120 healthy participants were divided into three groups: the whole-body electrostimulation group, the local electrostimulation group, and the control group without electrical stimulation. All groups performed a single session with the same dynamic exercise protocol. Muscle thickness and inter-rectus distance were evaluated ultrasonographically using the Rehabilitative Ultrasound Imaging technique both at rest and in muscle contraction (the active straight leg raise test) to find the post-intervention differences. The results showed significant differences in immediate post-intervention heart rate, with a smaller increase in the local electrostimulation group compared to the control and whole-body electrostimulation groups. No significant differences were identified between the groups after the interventions in the rest of the variables analyzed. Therefore, a local application, with the same effects as a global application on the abdominal musculature, has fewer contraindications, which makes its use more advisable, especially in populations with cardiorespiratory disorders, for which more research is needed.

## 1. Introduction

Electrical muscle stimulation (EMS) consists of the application of electrical currents to produce a visible muscle contraction [1]. Muscle strength gain can be achieved through EMS or physical exercise, but, according to the evidence, better results are obtained when both are performed together [1]. The application of EMS in conjunction with physical exercise is called dynamic EMS.

The application of dynamic EMS can be local or global [2]. Local EMS is applied to a specific body area, stimulating only the nerve or muscle cells in that area. The electrical current circulates locally between the positive and negative poles, located in the same muscle belly [3]. Less current is needed to cause a visible muscle contraction if the surface electrodes are placed at the motor point of the target muscle [4].

EMS can also be applied globally through whole-body electromyostimulation (WB-EMS), which allows a large muscular surface area (2800 cm^2^) [5] to be stimulated simultaneously, making it more functional training as it stimulates both agonists and antagonists [6]. The negative electrodes are placed on one half of the body and the positive electrodes on the other, with the electric current circulating between them and passing through the different tissues and organs of the body [3].

Both applications are well accepted even by untrained people, but they are not free of contraindications, especially when their application is global; in addition to the contraindications of EMS, there are systemic conditions (Diabetes mellitus, etc.) and health risks due to inappropriate use (rhabdomyolysis exertional) [7,8]. For this reason, the WB-EMS guide’s recommendations for its proper and safe use have been considered in this study [9].

Physical training with WB-EMS/EMS obtains similar results in muscle strength as conventional training but with a lower training intensity [10]. Therefore, this method can be considered an alternative [2] in populations that for various circumstances cannot perform a high-intensity physical exercise (advanced age, cardiovascular or respiratory pathologies, sarcopenia, sarcopenic obesity, menopausal women, etc.) [11] or for strengthening muscles that are more difficult to obtain maximum muscle contraction due to their biomechanical characteristics, such as the muscles of the anterolateral abdominal wall and especially of the deep musculature (Transversus Abdominis, TrA) [12].

The abdominal wall musculature (Internal Oblique (IO), External Oblique (EO), and rectus anterior (RA), TrA) plays a fundamental role in the stabilization of the pelvis and lumbar spine. Due to its anatomical peculiarities and functional complexity [13], more research is needed on this body region with the use of modern techniques, such as real-time ultrasound [14]. Rehabilitative Ultrasound Imaging (RUSI) is a non-invasive, objective, and validated technique to evaluate structural muscle changes and their behavior [15]. It has high reliability in the measurement of trunk muscles [16,17] and has been used in various investigations on the effects of EMS on the abdominal musculature [15,18,19,20,21].

The application of EMS to the abdominal wall, in addition to improving muscle strength, produces preferential stimulation of the central stabilizing musculature over the superficial musculature [19]. Indeed, patients who suffered from low back pain presented a loss of automatic control of deep musculature, as shown by TrA showing ultrasound thickness and electromyography muscle activity reductions during isometric leg tasks compared to healthy participants [22]. However, these benefits are not so clear in healthy populations [23,24].

There are several studies on the chronic application of dynamic WB-EMS, especially on the limb musculature and its effects on physiological parameters [2,6,25,26,27,28,29,30], while studies on local EMS are mostly not combined with physical exercise [31]. Few studies analyze the evolution of morphological and physiological changes during a period of training, and there may be differences depending on the type of muscles stimulated [32]. More research is needed on the effects of dynamic EMS [33]. The scientific literature is scarce on the immediate effects of a single WB-EMS/EMS session in healthy individuals, especially on the abdominal musculature, and an objectively assessing the immediate changes in its thickness using RUSI and its acute effects on physiological parameters such as heart rate. Likewise, we have detected a scarcity of studies evaluating the possible differences between local (local EMS) and global (WB-EMS) applications.

According to Coghlan et al. [19], a single session of EMS may preferentially promote the stimulation of deep stabilizer abdominal muscle contraction, increasing muscle thickness by RUSI, and was claimed as a potential therapeutic intervention, although the immediate effects of WB-EMS over the abdominal musculature remain unknown. Thus, our alternative hypothesis was that the application of dynamic EMS would cause immediate changes in abdominal muscle morphology, or IRD, with respect to WB-EMS or control groups, as well as different changes in physiological parameters in both experimental groups compared to the control group. Nevertheless, the null hypothesis was that the application of WB-EMS or dynamic EMS would not cause immediate changes in abdominal muscle morphology, IRD, or physiological parameters compared to the control group. Lastly, the aim of this study was to identify and compare the acute effects of a dynamic exercise session with WB-EMS and local EMS on the thickness of the abdominal wall musculature (TrA, IO, EO, and RA) and the inter-rectus distance (IRD) in young, healthy individuals as evaluated by ultrasound. Additionally, as a secondary objective, observe the immediate effects on physiological parameters: heart rate (HR), blood pressure (BP), and body temperature.

## 2. Materials and Methods

### 2.1. Study Design

A parallel-group, triple-blind, randomized controlled trial (participants, evaluator, and statistician) was performed between November 2021 and June 2022, following the CONSORT 2010 criteria [34] (the Consolidated Standards of Reporting Trials statement). The protocol for this study was prospectively registered on Clinical Trials.gov (ID: NCT05117203) and approved by the Ethics Committee of the Universidad de León (code: ETICA-ULE-009-2020). Ethical regulations, as well as the Helsinki Declaration of Helsinki [35], the Spanish Law for Protection of Data (Organic Law 3/2018), and Biomedical Research in Human Participants (14/2007), were respected. The standard informed consent procedure was followed and signed by all subjects who agreed to participate in this study.

### 2.2. Participant Recruitment

Students from the Faculty of Health Sciences of the Ponferrada Campus (Universidad de León, Ponferrada, León, Spain) were recruited by probability sampling through a publicity campaign by the investigators. The inclusion criteria for the study were: (a) healthy subjects of both sexes; (b) age range between 18 and 35 years old; (c) a good medical history with normal medical examinations and no previous history of cardiac disturbance; and (d) no surgeries in the previous year. The exclusion criteria were [8,9]: (a) body mass index (BMI) greater than 30 kg/m^2^; (b) elite or professional physical activity performance; (c) hyperventilation/hypercapnia and a score equal to or greater than 23 points on the Nijmegen questionnaire [36]; (d) women during their menstrual period; (e) habitual intake of medications; (f) abdominal surgeries (scars or keloids); (g) old or recent muscle injury at the abdominal level; (h) congenital diseases with musculoskeletal alterations at the level of the back and lower extremities, such as scoliosis, protrusion, or disc herniation; (i) presence of chronic low back, hip, or thigh pain; and (j) present any contraindication to WB-EMS/EMS [7]: pregnancy; viral or bacterial infections; arterial circulatory disorders, advanced arteriosclerosis; type I diabetes; hemophilia, bruising, hemorrhage; cognitive deficits; neuronal diseases, neuronal disorders, or epilepsy; recently performed operations in stimulation areas; abdominal wall and inguinal hernia; acute diseases, or inflammatory diseases, etc.

### 2.3. Sample Size Calculation

Sample size calculation was carried out by the software G*Power 3.1.9.2. using the F-test family for fixed effects, omnibus, and one-way analysis of variance (ANOVA). Based on a previous pilot study (*n* = 15) divided into 3 groups with 5 participants per group, using the mean of the difference (post-pre) of the thickness change during muscle activation (contraction-rest) in the TrA for EMS group (0.028 cm), the WB-EMS group (−0.006 cm), and the control group (0.018 cm) were calculated, as well as the standard deviation (SD) within each group (0.048). An effect size f of 0.294 was used for the sample size calculation in conjunction with an α error probability of 0.05, a power (1 – β error probability), and 3 for the number of groups, obtaining a necessary total sample size of 117 participants, divided into 39 subjects per group, in order to achieve an actual power of 0.809.

### 2.4. Randomization and Blinding

Triple-blind study (participants, evaluator, and statistician). The intervention investigator performed randomization using sealed, opaque envelopes that determined the intervention group [37] and assigned a numerical code to each participant generated with computer software. The ultrasound evaluation investigator and the statistician received the blind-coded data from the study groups. Sham treatment participants were blinded to their membership in the control group.

### 2.5. Procedure

The study began in November 2021 with an initial contact with each participant to record their demographic data, anthropometric measurements, and level of physical activity using the International Physical Activity Questionnaire (IPAQ-SF) [38] and the Nijmegen questionnaire [36], in addition to performing an anamnesis to verify the absence of any contraindication for the use of WB-EMS/EMS or any exclusion criteria for this study. Finally, the correct execution of the dynamic exercises and the Borg scale [39] were explained to them. A series of guidelines on the use of WB-EMS and anti-COVID-19 measures were recommended for an adequate and safe intervention.

**Figure 1:**
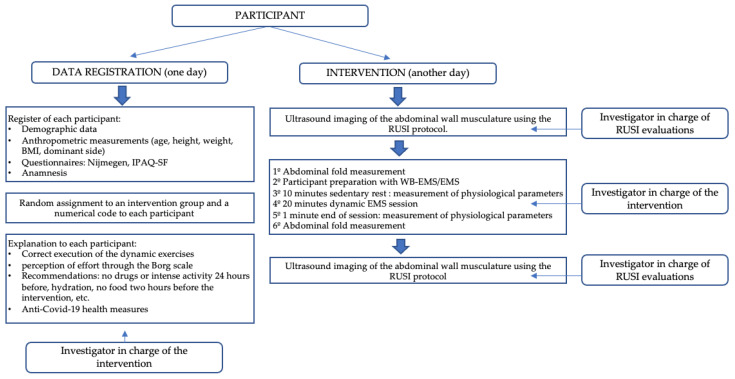
Diagram of the intervention procedure.

According to Figure 1, the pre-protocol data collection was performed after 10 min of resting in the sitting position before interventions assessing physiological parameters, the abdominal fold, and RUSI measurements [25,26].

Next, the interventions were performed by the same researcher who received previous training on WB-EMS/EMS [9,40], and they started in January 2022, in the afternoon hours, at the same location and under the same environmental conditions (22°–24° ambient temperature and 40–60% relative air humidity). Please see Section 2.6 below for detailed interventions.

Finally, only one post-protocol collection time was applied after 1 min of resting in the sitting position after interventions for physiological parameters, abdominal fold, and RUSI measurements [25,26]. The RUSI protocol was carried out by the same researcher in charge of capturing the RUSI images and measurements, with experience in ultrasound evaluation of the abdominal musculature and prior training on the RUSI protocol, in a different room to the dynamic EMS intervention to ensure its blinding. Please see Figure 1 and Section 2.7 below for detailed ultrasound evaluations.

### 2.6. Interventions

The intervention consisted of a single session of a dynamic exercise aimed at stimulating the contraction of the abdominal musculature combined with local or global EMS (Table 1), supervised by the responsible researcher, ensuring the safety of the participants [9,41].

The guide of recommendations for the appropriate and safe use of WB-EMS [9] was considered, applying an intervention protocol with safe electrical parameters for the participant based on scientific evidence [2].

The dynamic exercises were coordinated with the electric current by performing the gesture in the impulse phase and returning to the initial position in the rest phase [42].

**Table 1:** Dynamic EMS session protocol (electrical stimulation parameters) [41,43,44].

Session: 20’	WB-EMS/EMS	Dynamic Exercise Protocol [41,43,45]
**WARM UP** **5’**	Bipolar rectangular current 20 Hz—350 µs No periods of stimulation/rest 2/3 RPE	Walking, running, and skipping
**MAIN PART** **12’**	Bipolar rectangular current 85 Hz—350 µs 4″ stimulation/4″ rest 4/5/6 RPE	1 series × 8 repetitions: dynamic squats; 2 series × 8 repetitions: dynamic side lunges (right–left); 2 series × 8 repetitions: lateral trunk flexion; 2 series × 8 repetitions: static forward lunges (right–left); 1 series × 8 repetitions: fitball tilting; 1 series × 8 repetitions: fitball front plank; 2 series × 8 repetitions: dynamic crunches diagonally (right–left); 2 series × 8 repetitions: side plank right and left.
**COOL DOWN** **3’**	Bipolar rectangular current 5 Hz—150 µs 1″ stimulation/1″ rest 2/1 RPE	Stretching

WB-EMS group: Exercise intervention with WB-EMS (Justfit; https://justfitart.com accessed on 1 February 2023) with wireless sensors controlled by Bluetooth technology via a tablet. Nine muscle groups were activated simultaneously: arms, trapezius, dorsal, lumbar, gluteus, quadriceps, femoral, abdomen, and the electrodes of the pectoral area were placed on the sides of the abdominal wall. The intensity was adjusted individually for each muscle group. The participants wore cotton clothing previously moistened with water as well as electrodes [46].

EMS group: Exercise intervention with EMS in abdominal muscles (PhysiomedExpert, Physiomed Elektromedizin AG; a variable intensity maximum of 75 mA at 500 ohms of impedance and 230 volts at the maximum voltage peak). Rectangular adhesive electrodes (100 × 50 mm) were placed on the TrA and lateral wall (pencil electrode to locate skin areas of best response to electrical stimulation) [4].

Control Group: Exercise intervention with WB-EMS without electrical stimulation [45]. The same protocol as the WB-EMS Group was followed, with individual adjustment of the current intensity in each muscle group so that they would perceive the electric current (blinding belonging to the control group), lowering the intensity to zero at the beginning of the dynamic exercise session.

To achieve the appropriate intensity of the application with WB-EMS/EMS dynamically [5], each participant’s rating of perceived exertion (RPE) using the modified Borg scale CR-10 [39] was used, with participants perceiving it as “somewhat strong” and “strong” in the main part of the session (4–5 on the Borg CR-10 Scale). Depending on the tolerance level of each participant, the intensity was increased every 3 min without exceeding level 6 to avoid possible negative consequences of high exertion intensity without prior adaptation to the WB-EMS [46].

In the warm-up phase, the intensity was 2/3 RPE (from light to moderate), and in the cool-down phase, it was 2/1 RPE (from light to very light).

The intensity of the dynamic exercises in the control group, with the WB-EMS but without electrical stimulation, was also adjusted with RPE with the same parameters as dynamic EMS.

### 2.7. Ultrasound Protocol

An ultrasound tool (Versana ActiveTM, General Electric; GE HealthCare, Madrid, Spain) with a linear probe using a trapezoidal preset to expand the scanning area and a 5–13 MHz range (12L-RS type) [40] was used to generate ultrasound images in B-mode; the imaging measurements were performed in the ultrasound tool’s software.

**Figure 2:**
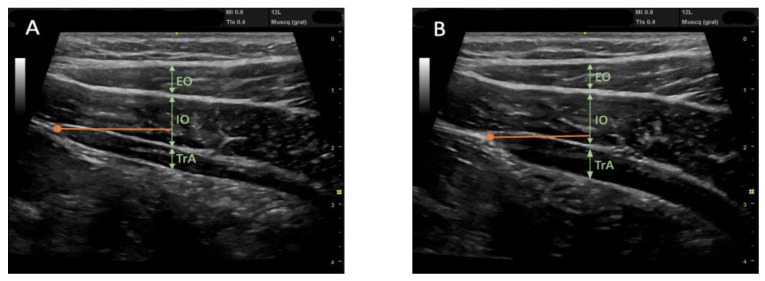
Ultrasound images of the lateral abdominal wall musculature showing the 3 muscle layers from top to bottom: External Oblique (EO), Internal Oblique (IO), and the deepest, Transverse Abdomen (TrA); separated from each other by intermuscular connective tissue (hyperechoic lines). (**A**) Image at rest. (**B**) An image taken during muscle activity showing a change in muscle thickness. Measurements are always taken at the same point, 2 cm (the orange mark) from the insertion of the TrA in the RA.

**Figure 3:**
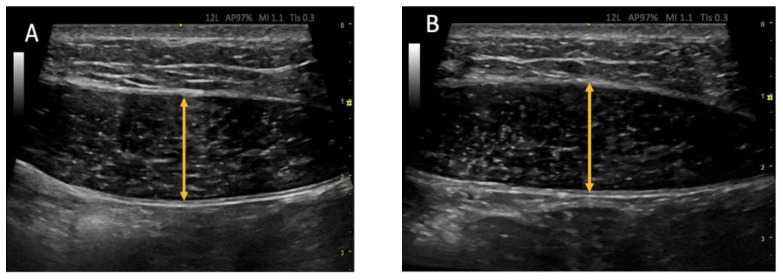
Ultrasonic images of the rectus abdominis (RA). (**A**) at rest and (**B**) during muscle activity. Measurements are taken between the internal borders of the widest area of the muscle belly. An increase in muscle thickness is appreciable during contraction (**B**).

**Figure 4:**
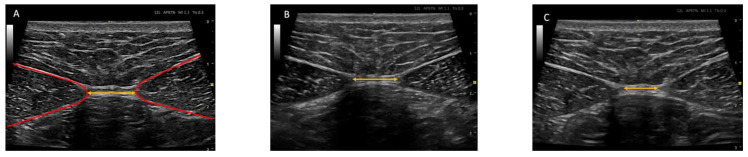
Ultrasound images of the rectus abdominis distance (IRD). (**A**) Right and left abdominal rectus abdominis (areas between the red lines) and intermediate fascia corresponding to the IRD (represented by the yellow line). (**B**) Ultrasound image of the IRD at rest. (**C**) Ultrasound image of the IRD during muscle activity.

The RUSI technique was used bilaterally to assess abdominal wall muscle thickness at rest and during muscle activation (contraction—rest thickness) [47,48]. The thickness of the abdominal wall musculature (TrA, IO, EO, and RA) was measured between the inner limits of each muscle at rest and during muscle activity (Figure 2 and Figure 3) (CCI between 0.62 and 0.99 for muscle thickness and between 0.48 and 0.78 for the comparison of the change in muscle thickness concerning the resting basal value) [49]. The IRD was measured between the inner limits of the medial borders of both RA at rest and during muscle activity (Figure 4); the CCI was between 0.74 and 0.90 [50].

**Figure 5:**
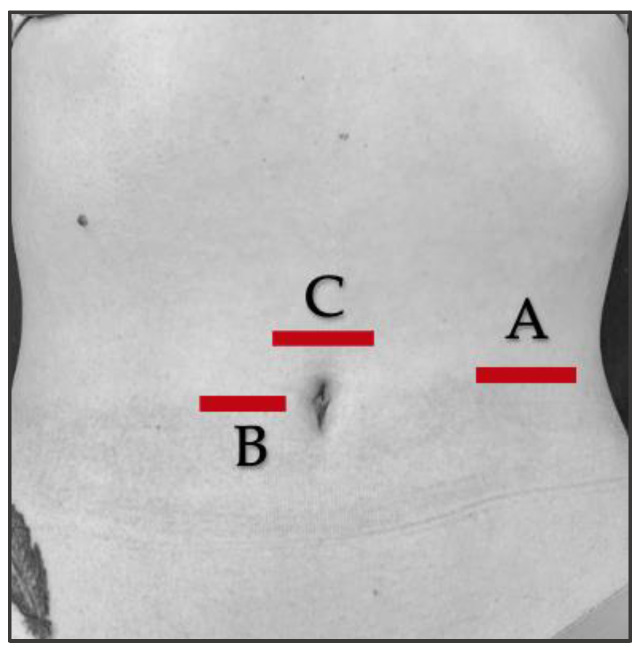
Reference points for the RUSI images [47,50]. (A) Midway between ASIS (antero superior iliac spine) and the last rib, on the midaxillary line, to obtain images of the TrA, IO, and EO muscles. A horizontal line was drawn 2 cm lateral to the TrA insertion in the RA connective tissue as a reference for TrA, IO, and EO measurements at rest and during muscle contraction; (B) Midway along the RA muscle at the level of the umbilicus, transverse to acquire the RA image. The reference point for RA thickness measurement was the mean distance of the width of each RA; (C) In the middle of the abdomen, a distance of 2 cm above the umbilicus to measure IRD.

The position of the participants was supine decubitus with a neutral position of the upper and lower extremities [51]. The probe was placed transversely to the abdominal wall [47] for RUSI assessments at rest and during muscle activity, without exerting pressure on the skin, and held in the same location and with the same pressure (only the weight of the probe itself) at each reference point (Figure 5).

To assess abdominal wall muscle contraction, the active straight leg raising test (aSLRT) was used to activate the abdominal musculature (CCI of 0.65–0.69 for change in TrA thickness and 0.65–0.79 for OI) [51]. The participant was asked to actively raise the leg on the side to be assessed from the couch, with the knee straight, 30° of hip flexion (measured with a universal goniometer) [51], or 15 cm from the starting position.

RUSI imaging was performed before and after each intervention. The evaluator explained the RUSI procedure to the participant beforehand and gave the following execution commands [18]: “prepare to lift”, “lift”, “hold for 10 s of lifting”, “prepare to lower”, and “lower” for the return to the starting position.

At each set point, three ultrasound images were captured for reliability: during rest and aSLRT [18], at the end of unforced expiration [48], and with 30″ rests between each to minimize the influence of muscle fatigue [18]. The order of measurements was randomized before RUSI assessments to reduce potential measurement bias.

### 2.8. Outcome Measurements/Descriptive Data

Outcome measures were recorded at baseline and at the end of the intervention.

The outcome measurements were TrA muscle thickness (main outcome measurement), IO, and EO as the IRD, which were assessed at rest and during aSLRT to calculate their thickness changes (aSLRT-rest thickness difference), as well as HR, BP, and temperature (Visomat comfort 20/40 sphygmomanometer; Uebe Medical GmbH, Zerbst, Germany; accuracy clinically validated by the European Society of Hypertension).

The descriptive data were: age (years); sex (female/male); body weight (kg); body height (m); BMI (kg/m^2^) according to the Queletet method [52]; respiratory distress measured by the Nijmegen questionnaire (Spanish version) (specificity of 0.91 and sensitivity of 0.95 to detect the presence of hyperventilation or hypercapnia that can alter the function of the TrA) [36]; and level of physical activity measured by the International Physical Activity Questionnaire, short Spanish version (IPAQ-SF) (low, moderate, and high levels) (adequate reliability from 0.66 to 0.88) [38].

### 2.9. Statistical Analyses

Statistical analyses were carried out using the 22.0 version of the Social Sciences Statistical Package (SPSS) software. Normal distribution was assessed by the Kolmogorov–Smirnov test. Categorical data were described by frequency (*n*) and percentage (%), and their comparison was performed by the chi-square test. Quantitative data adjusted for normal distribution were described by mean ± standard deviation (SD) and completed with range (minimum–maximum), and their between-groups comparison was performed using one-way analysis of variance (ANOVA). Quantitative data adjusted for non-normal distribution were described by median ± interquartile range (IR) and completed with range (minimum–maximum), and their between-groups comparison was performed using the Kruskal–Wallis test. Effect size for F-tests was determined by the partial Eta squared coefficient (ηp^2^), interpreting ηp^2^ = 0.01 as a small effect size, ηp^2^ = 0.06 as a medium effect size, and ηp^2^ = 0.14 as a large effect size [53,54,55]. Post-hoc comparisons were performed using Bonferroni’s correction and adjusted *p*-values, as well as their effect sizes, which were calculated by Cohen’s *d* and categorized as very small effect sizes if *d* < 0.20, small effect sizes if *d* = 0.20–0.49, medium effect sizes if *d* = 0.50–0.79, and large effect sizes if *d* > 0.8 [56]. *p*-values < 0.05 were interpreted as statistically significant regarding a 95% confidence interval (CI).

In order to detail intra- and intergroup comparisons, a 2-way analysis of variance (ANOVA) was performed, including 3 groups and 2 measurement moments, considering repeated measurements across time (before and after interventions) as a within-subject factor as well as groups (EMS, WB-EMS, and control groups) as a between-group factor, and completed with linear graphs in order to detail comparisons for all outcome measurements, respectively [57]. Furthermore, the significance of these tests was considered by the Greenhouse–Geisser correction when the Mauchly tests rejected sphericity [58]. Indeed, Bonferroni’s corrections were applied to determine post-hoc comparisons. Again, effect sizes for F-tests were calculated by the partial Eta squared (ηp^2^) coefficients, as described previously [53,54,55].

Finally, multivariate regression analyses were performed to predict the outcome measurement differences (post-pre) after intervention based on baseline data to check if baseline differences or characteristics could influence our study results. Linear regression models were performed by the stepwise selection method, and the *R*^2^ coefficient was calculated to determine the adjustment quality [59]. Baseline data were selected as independent variables, including group (EMS = 1; WB-EMS = 2; and control = 3), sex (male = 1; female = 2), dominance (right = 1; left = 2), age (years), IPAQ (METs/min/week), sitting time (minutes), IPAQ level (sedentary = 1; moderate = 2; and vigorous = 3), Nijmegen score (points), weight (kg), height (m), BMI (kg/m^2^), and abdominal fold (mm). Outcome measurements differences (post-pre) after the intervention were selected as the dependent variables. Pre-established F-probabilities values from Pin = 0.05 to Pout = 0.10 were considered.

## 3. Results

### 3.1. Flow Diagram

**Figure 6:**
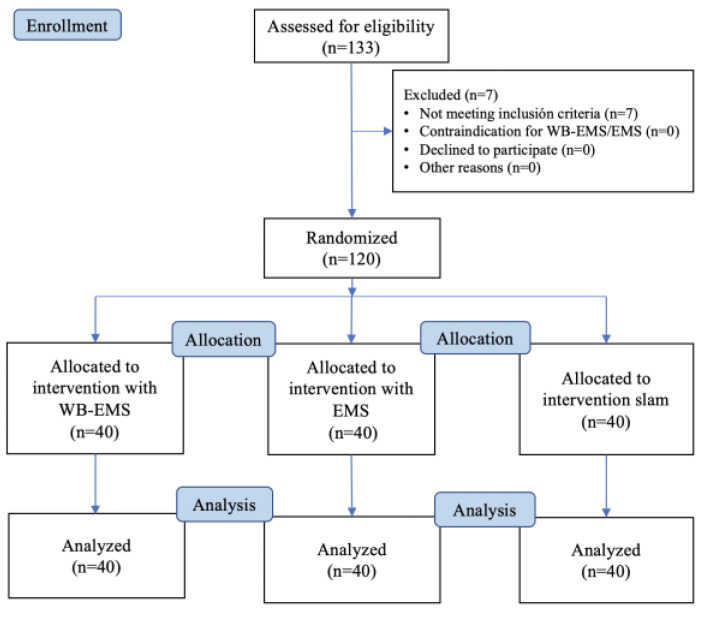
Flow diagram including all phases of the randomized clinical trial [34].

All participants assessed for eligibility (*n* = 120) completed the study course and were randomized into the EMS (*n* = 40), WB-EMS (*n* = 40), and control (*n* = 40) groups. Seven participants were excluded for the following reasons: abdominal surgery in the last year (*n* = 1); recent muscle injury (*n* = 1); score >23 points on the Nijmegen questionnaire (*n* = 3); BMI > 30 kg/m^2^ (*n* = 1); and professional sports activity (*n* = 1) (Figure 6).

### 3.2. Baseline Measurements

Descriptive data comparisons did not show statistically significant differences (*p* > 0.05) among the EMS, WB-EMS, and control groups for IPAQ score, Nijmegen score, height, BMI, sex, dominance, and IPAQ level (Table 2). Nevertheless, there were statistically significant differences for age distribution (*p* = 0.033), sitting time (*p* < 0.001), abdominal fold (*p* < 0.001), and dominance (*p* < 0.001). According to Bonferroni’s correction, post-hoc comparisons showed older age for the WB-EMS group with respect to the control group (*p* = 0.035), longer sitting time for the EMS group with respect to the WB-EMS (*p* < 0.001) and control (*p* = 0.001) groups, as well as greater abdominal folds for the EMS group with respect to the WB-EMS (*p* = 0.017) and control (*p* < 0.001) groups. In addition, the EMS groups showed a higher presence of left dominance with respect the WB-EMS and control groups. The rest of the post-hoc comparisons did not show statistically significant differences (*p* > 0.05).

Comparisons for outcome measurements at baseline did not show any statistically significant difference (*p* ≥ 0.05) among the EMS, WB-EMS, and control groups for HR, SBP, DBP, and temperature, as well as bilaterally for TrA, RA, IO, and EO muscle thickness and IRD changes after interventions (Table 3).

### 3.3. Outcome Measurements Differences after Interventions

**Figure 7:**
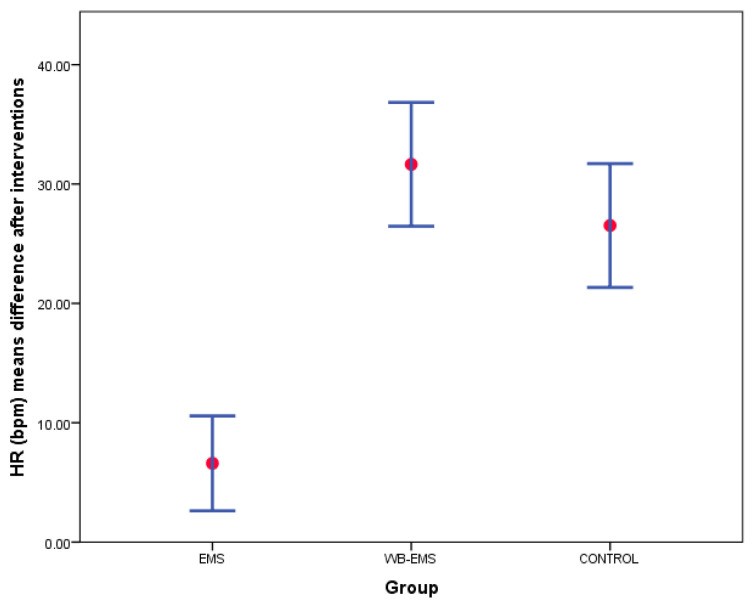
Bar graphs showing HR mean differences after interventions completed with SD among the EMS, WB-EMS, and control groups. Abbreviations: bpm, beats per minute; EMS, electrical muscle stimulation; SD, standard deviations; WB-EMS, whole body electrical muscle stimulation.

Despite comparisons for outcome measurement differences after interventions showing no statistically significant differences (*p* > 0.05) among EMS, WB-EMS, and control groups for SBP, DBP, and temperature, as well as bilaterally for TrA, RA, IO, and EO muscles thickness and IRD changes after interventions (Table 4), there were between-groups statistically significant differences with a large overall effect size for HR differences after interventions (*p* < 0.001; F_(2,117)_ = 30.874; ηp^2^ = 0.345). According to Bonferroni’s correction, post-hoc comparisons showed an HR increase with a large effect size for the WB-EMS (mean difference = 25.05 bpm; *p* < 0.001; d = 1.53) and control (mean difference = 19.92 bpm; *p* < 0.001; d = 1.22) groups with respect to the EMS group (Figure 7).

### 3.4. Two-Way ANOVA of Repeated Measurements for Intra- and Intergroup Comparisons

**Figure 8:**
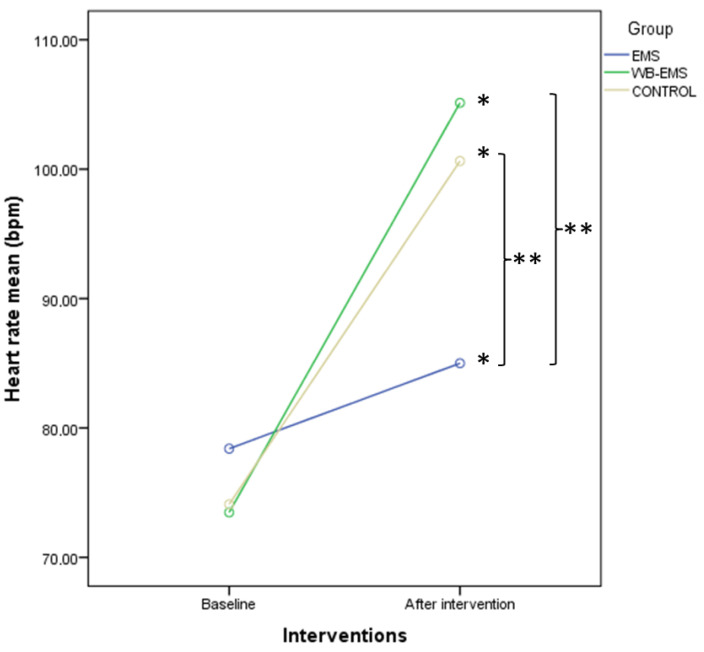
Linear graphs showing HR means before and after interventions as well as intra- and intergroup comparisons among the EMS, WB-EMS, and control groups. Abbreviations: bpm, beats per minute; EMS, electrical muscle stimulation; WB-EMS, whole body electrical muscle stimulation. * Intragroup comparisons showed statistically significant differences (*p* < 0.05). ** Intergroup comparisons showed statistically significant differences (*p* < 0.05).

The described findings were confirmed by the two-way ANOVA for repeated measurements to detail intra- and intergroup comparisons. Firstly, HR showed significant differences for time (*p* < 0.001; F = 246.546; ηp^2^ = 0.678) and time*group interaction (*p* < 0.001; F = 30.874; ηp^2^ = 0.345). Post-hoc comparisons showed intragroup statistical differences (*p* < 0.01) for a HR increase in all groups after interventions and intergroup statistical differences with a large effect size (*p* < 0.001; *d* = 1.22–1.53) for a HR increase in both the WB-EMS and control groups with respect to the EMS group (Figure 8).

**Figure 9:**
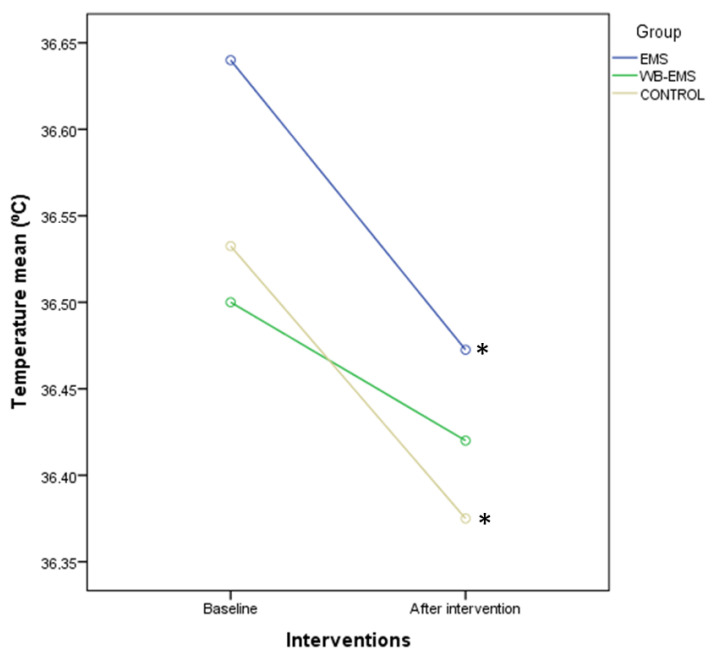
Linear graphs showing temperature means before and after interventions as well as intragroup comparisons among the EMS, WB-EMS, and control groups. Abbreviations: EMS, electrical muscle stimulation; WB-EMS, whole body electrical muscle stimulation. * Intragroup comparisons showed statistically significant differences (*p* < 0.05).

Furthermore, the ANOVA for repeated measurements of SBP did not show significant differences for time (*p* = 0.246; F = 1.362; ηp^2^ = 0.012) or time × group interaction (*p* = 0.312; F = 1.177; ηp^2^ = 0.020). In addition, DBP did not present significant differences for time (*p* = 0.342; F = 0.911; ηp^2^ = 0.008) and time*group interaction (*p* = 0.439; F = 0.829; ηp^2^ = 0.014). Next, an ANOVA for repeated measurements of temperature showed significant differences for time (*p* = 0.002; F = 9.667; ηp^2^ = 0.076), but not for time × group interaction (*p* = 0.668; F = 0.406; ηp^2^ = 0.007). Intragroup comparisons by Bonferroni’s corrections showed a significant reduction of the temperature after EMS (*p* = 0.028; *d* = 0.21) and control (*p* = 0.038; *d* = 0.42) interventions, but not after WB-EMS (*p* = 0.290; *d* = 0.21) interventions (Figure 9).

**Figure 10:**
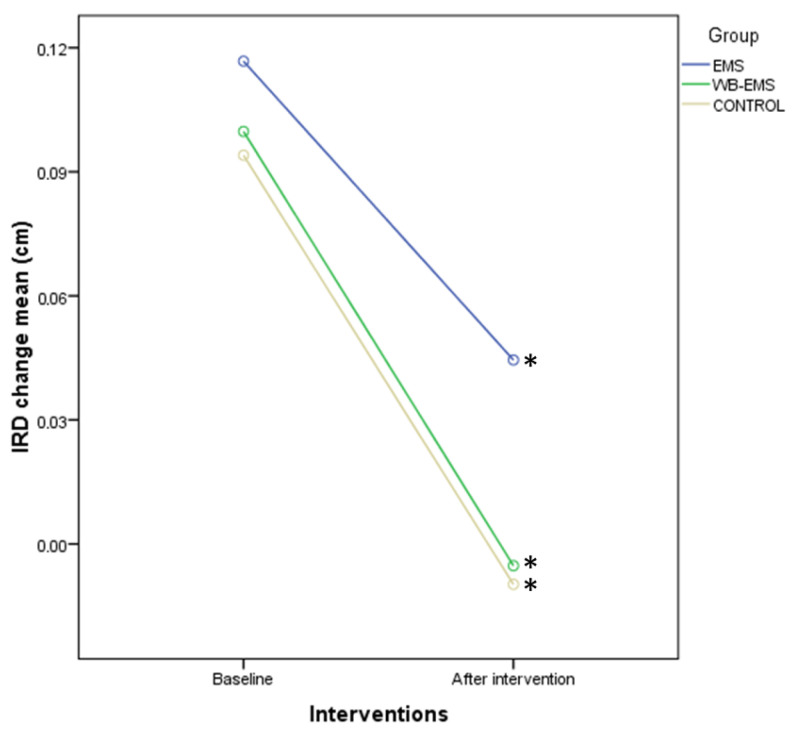
Linear graphs showing IRD change before and after interventions as well as intragroup comparisons among the EMS, WB-EMS, and control groups. Abbreviations: cm, centimeters; EMS, electrical muscle stimulation; IRD, inter-rectus distance; WB-EMS, whole body electrical muscle stimulation. * Intragroup comparisons showed statistically significant differences (*p* < 0.05).

In addition, IRD change presented significant differences for time (*p* < 0.001; F = 32.877; ηp^2^ = 0.219), although not for time*group interaction (*p* = 0.651; F = 0.430; ηp^2^ = 0.007). Indeed, intragroup comparisons using Bonferroni’s correction displayed a significant reduction of the IRD change after all interventions, such as EMS (*p* = 0.012; *d* = 0.37; *d* = 0.37), WB-EMS (*p* < 0.001; *d* = 0.63), and control (*p* < 0.001; *d* = 0.97) groups (Figure 10).

Regarding the rest of the RUSI measurements, there were not statistically significant differences for time considering the thickness changes of the right (*p* = 0.942; F = 0.005; ηp^2^ = 0.000) and left (*p* = 0.466; F = 0.536; ηp^2^ = 0.005). TrA, right (*p* = 0.618; F = 0.250; ηp^2^ = 0.002), and left (*p* = 0.566; F = 0.331; ηp^2^ = 0.003). IO, right (*p* = 0.363; F = 0.835; ηp^2^ = 0.007) and left (*p* = 0.081; F = 3.093; ηp^2^ = 0.026) EO, as well as the right (*p* = 0.975; F = 0.001; ηp^2^ = 0.000) and left (*p* = 0.971; F = 0.001; ηp^2^ = 0.000) RA. Likewise, there were not statistically significant differences for time × group interaction regarding thickness changes on the right (*p* = 0.891; F = 0.115; ηp^2^ = 0.002) and left (*p* = 0.415; F = 0.885; ηp^2^ = 0.015). TrA, right (*p* = 0.769; F = 0.263; ηp^2^ = 0.004) and left (*p* = 0.769; F = 0.263; ηp^2^ = 0.004) IO, right (*p* = 0.671; F = 0.400; ηp^2^ = 0.007) and left (*p* = 0.792; F = 0.234; ηp^2^ = 0.004) EO, and right (*p* = 0.190; F = 1.683; ηp^2^ = 0.028) and left (*p* = 0.322; F = 1.145; ηp^2^ = 0.019) RA.

### 3.5. Multivariate Linear Regression Models

Multivariate regression analyses did not display any valid regression model to predict the outcome measurement differences after interventions for HR, SBP, and DBP, as well as IRD and thickness changes of the left TrA and bilaterally the IO and EO muscles. Nevertheless, a linear regression model (*p* = 0.014; F_(1,118)_ = 6.275; R^2^ = 0.050; β = +0.003) showed that a greater right TrA thickness change difference after intervention was predicted by a higher Nijmegen test score. In addition, a linear regression model showed that a lower right RA thickness change difference after intervention (*p* = 0.008; F_(1,118)_ = 7.238; R^2^ = 0.058; β = −0.150) was predicted by lower height of participants, as well as another linear regression model (R^2^ = 0.123), which determined that a lower left RA thickness change difference after intervention was predicted by lower height (*p* = 0.005; F_(1,118)_ = 8.330; R^2^ = 0.066; β = −0.271) and Nijmegen scores (*p* = 0.007; F_(1,118)_ = 9.606; R^2^ = 0.057; β = −0.003). Finally, a linear regression model (*p* = 0.002; F_(1,118)_ = 6.275; R^2^ = 0.075; β = +0.262) displayed that a higher temperature difference was predicted by female sex. Thus, statistically significant between-group differences were shown at baseline (Table 2), such as age, sitting time, abdominal fold, and dominance, which did not influence nor predict the outcome measurement differences.

## 4. Discussion

The main objective of this study was to identify the effects of a single session of dynamic exercises with WB-EMS or EMS on the thickness of the abdominal wall musculature. The results did not show significant changes in the morphology of the deep (TrA and IO) and superficial (RA and EO) abdominal musculature compared to the control group; therefore, the hypothesis raised in this research is confirmed.

To our knowledge, this is the first study to investigate the immediate effects of a session of local EMS and WB-EMS on the abdominal muscles in healthy young people, assessed using RUSI.

No significant differences were obtained in the abdominal muscle thickness of the WB-EMS/EMS interventions versus the control group with WB-EMS deactivated, despite several studies suggesting that the application of EMS can lead to greater muscle metabolic stress (oxygen consumption (VO2), lactate and hormone levels, and delayed onset muscle soreness (DOMS)) [23,28] than voluntary muscle contraction by causing greater muscle fiber recruitment (synchronous) [11], leading to higher acute energy expenditure during exercise compared to exercise alone [60] and a higher level of muscle fatigue, especially with global application with WB-EMS, as it stimulates a larger body surface area than local EMS [61].

The acute effect of increased muscle thickness observed in other studies [62] may be due to an increase in muscle protein synthesis resulting in “muscle swelling” or to an acute inflammatory response induced by exercise, immediately after the first training session [62] and especially with the use of EMS currents. This acute inflammatory response depends on the volume, intensity, type of exercise, and level of fatigue [63], which is an indirect marker of muscle damage. High-intensity or high-volume endurance or strength training leads to greater acute responses in muscle thickness and greater changes in muscle morphology, especially due to high volume [64], which is not the case with our protocol.

The acute muscle response visible with ultrasound has been similar in the three interventions, suggesting that the dynamic exercise protocol [62,64] and the electrical current parameters of this study are safe, as in previous studies that used those parameters with different types of populations [3] and recorded no adverse reactions [65].

The sample in this study was healthy; no differences were obtained in the acute effects of the addition of WB-EMS/EMS to the physical exercise protocol, as in other studies with healthy populations that did not find a greater benefit with EMS training [9,29,30]. In contrast, in populations with certain pathologies (low back pain and abdominal rectus diastasis), better results were obtained on the abdominal musculature in terms of muscle mass gain, muscle strength, and improved abdominal muscle recruitment with the combination of EMS and physical exercise [20,23,66].

Based on our findings, a single session of dynamic WB-EMS/EMS did not generate a sufficient stimulus to produce a visible muscle response in the abdominal musculature. Despite the parameters used (bipolar rectangular, 85 Hz, 350 µsg, and 4″ of impulse and 4″ of rest) being sufficient to develop a high voluntary muscle contraction [41,67], they did activate the necessary muscle responses leading to strength adaptations [67].

Previous studies [32,68] that examined the temporal evolution of muscular changes at a morphological level during a training period found that in the first sessions, muscular responses are produced at a molecular level, stimulating processes of myofibrillar protein synthesis [68], which are not detectable with the ultrasound measuring instrument used in this study.

These muscular responses added over time give rise to morphological changes at least, with 4 weeks of acute sessions of strength exercise with EMS [68] becoming evident in the increase in muscle thickness within the first 6 weeks [20]. Long-term EMS produces muscle hypertrophy, with 8 weeks of strength or resistance training being necessary [30,66,69], with adaptations in muscle mass and architecture occurring between the 4th and 8th weeks [32].

Furthermore, no changes were seen in the thickness of the abdominal musculature according to the dominant or non-dominant side or in the muscle response to EMS as a function of abdominal crease thickness. Other studies in healthy individuals also did not observe differences in thickness in abdominal musculature on both sides, both at rest and during muscle contraction [14,21].

In terms of physiological parameters, no significant immediate effects were obtained for BP or body temperature with any of the three interventions compared to their baseline values before the session. On the other hand, a different behavior was observed in the HR after the application of local EMS compared to the other groups. The HR measured immediately at the end of the intervention increased in all three groups compared to the baseline HR, but its increase with local EMS was significantly lower than with the application of WB-EMS (+25 beats/minute compared to EMS) or with exercise alone (+19 beats/minute compared to EMS).

Electrical stimulation can modulate sympathetic and parasympathetic activity [70]. There are few studies evaluating acute HR modifications due to the application of dynamic WB-EMS/EMS in a single session; as in our results, they observed that the increase in HR immediately following dynamic exercise with WB-EMS was greater in both groups (obese and healthy) than with exercise alone, but the differences were not statistically significant. They concluded that WB-EMS did not alter cardiac autonomic modulation in the obese young population [25,26] or in the healthy [25,27].

The results obtained from the cardiac parameters analyzed suggest that it is a safe procedure, coinciding with the study of Jee (2018) [45], in terms of cardiopulmonary factors in healthy people with similar characteristics to our sample.

Post-intervention HR was higher with WB-EMS; this is because this device allows electrical stimulation of a larger body surface area than local EMS, stimulating several muscle groups simultaneously greater than the abdominal area, resulting in greater metabolic responses (lactate concentration), a higher level of muscle fatigue, and greater perceived exertion by the participant than local application or exercise alone [61].

The reasons why EMS reduces HR in terms of control may have different explanations. It could be because local EMS applied in the abdominal area enhances parasympathetic modulation of HR by an increase in its activity or by a decrease in sympathetic activity. Electrical stimulation may have produced a mild effect on vascular reflexes involving the autonomic nervous system, causing a decrease in efferent sympathetic impulses and reducing sympathetic activity; it may have stimulated arterial baroreceptors, producing inhibition of sympathetic activity [71]; or it may have produced a lower perception of work effort due to modifications in breathing patterns, by direct stimulation of this musculature that supports respiratory function, resulting in different cardiovascular effects than the other interventions at the same work intensity (the placement of electrodes in local EMS is more precise, allowing motor point stimulation of the deep abdominal musculature to be more comfortable to tolerate and maximizing spatial recruitment of motor units [4]; whereas WB-EMS stimulation is non-specific and can produce simultaneous direct stimulation of somatosensory afferent nerve fascicles that influence the participant’s perception of pain and exertion) [4,72] or by a different functioning of the muscle metaboreflex that increases parasympathetic tone causing a lower increase in HR [73]. A greater number of studies with a more exhaustive assessment of cardiorespiratory and biochemical parameters (O_2_ uptake, lactate, and phosphocreatine accumulation) would be necessary to provide more information and could offer more plausible explanations.

It is necessary to recognize the limitations of this study, among them the use of a single session of dynamic EMS, sufficient to determine its immediate effects, although it would be interesting to know its effects with an intervention of several sessions; the cross-group study design when investigating the acute effect of the intervention; the use of electromyography to measure muscle activation in addition to the evaluations of the changes in muscle thickness carried out with RUSI and the calculation of the percentage difference in muscle thickness; the inclusion of an intervention group with WB-EMS/EMS (without exercise), although the three intervention groups performed an identical exercise protocol, to determine their effects on muscle structure and physiological parameters versus physical exercise; and a WB-EMS/EMS without abdominal stimulation to observe post-exercise HR behavior. As strong points, the obtaining of a representative sample with a high number of ultrasound measurements and the comparison of the possible differences between the application of EMS or WB-EMS in the abdominal musculature.

## 5. Conclusions

The application of a single session of electrostimulation (local or global) does not produce immediate acute changes in the thickness of bilateral abdominal muscles or in the inter-rectus distance (analyzed with the RUSI technique), which seems to indicate that the dynamic EMS protocol of this study does not produce acute inflammatory effects in these structures.

In addition, a single EMS session (local or global) does not produce statistically significant pre- and post-intervention changes in the physiological variables of body temperature and systemic blood pressure.

In contrast, there were significant differences between the groups analyzed in terms of HR after the interventions. The EMS group showed a smaller increase in posterior HR compared to the WB-EMS and control groups.

It has an interesting clinical application, since local EMS, with the same effects as WB-EMS on the abdominal musculature, has fewer contraindications, which makes its use more advisable, especially in populations with cardiorespiratory disorders. More research is needed in this field.

## Figures and Tables

**Table 2:** Descriptive data comparisons among the EMS, WB-EMS, and control groups.

Baseline Data	EMS (*n* = 40)	WB-EMS (*n* = 40)	Control (*n* = 40)	*p*-Value
Age (years)	21.00 ± 2.00 (18–30)	21.00 ± 6.00 (18–35)	20.00 ± 2.75 (17–28)	**0.033** †
IPAQ (METs/min/week)	3022 ± 2064 (889–11,000)	2679 ± 3101 (66–7944)	1879 ± 4650 (33–14,493)	0.253 †
Sitting time (minutes)	480 ± 225 (180–720)	300 ± 285 (0–600)	360 ± 172 (0–720)	**<0.001** †
Nijmegen (score)	12.42 ± 6.08 (0–22)	9.85 ± 5.59 (0–22)	12.67 ± 5.23 (3–22)	0.050 *
Weight (kg)	69.96 ± 15.18 (45–106)	66.36 ± 12.18 (39.90–97.50)	64.30 ± 11.33 (44.40–108.60)	0.148 *
Height (m)	1.71 ± 0.17 (1.52–2.00)	1.66 ± 0.12 (1.50–1.87)	1.64 ± 0.11 (1.50–1.83)	0.166 †
BMI (kg/m^2^)	19.54 ± 5.23 (14.15–29.94)	19.86 ± 4.71 (13.30–26.35)	18.97 ± 2.74 (14.14–29.67)	0.358 †
Abdominal fold (mm)	15.00 ± 6.75 (4–30)	12.00 ± 16.09 (0–35)	10.17 ± 11.58 (0–41.67)	**0.017** †
Sex (male/female)	20 (50%)/20 (50%)	17 (42.55%)/23 (57.5%)	13 (32.5%)/ 27 (67.5%)	0.281 ‡
Dominance (right/left)	21 (52.5%)/19 (47.5%)	37 (92.5%)/3 (7.5%)	36 (90%)/4 (10%)	**<0.001** ‡
IPAQ level (Level I/II/III)	0 (52.5%)/20 (47.5%)/ 20 (47.5%)	6 (52.5%)/13 (47.5%)/ 21 (47.5%)	4 (52.5%)/19 (47.5%)/ 17 (47.5%)	0.103 ‡

Abbreviations: BMI, body mass index; EMS, electrical muscle stimulation; IPAQ, Physical Activity Questionnaire (level I—slight, level II—moderate, and level III—vigorous); METs/min/week, metabolic equivalents per minute per week; WB-EMS, whole body electrical muscle stimulation. * Mean ± standard deviation, and range (minimum–maximum) were used and compared by the one-way analysis of variance (ANOVA). † Median ± interquartile range, and range (minimum–maximum) were used and compared by the Kruskal–Wallis test. ‡ Frequency (*n*) and percentage (%) were used and compared by the chi-square test. *p*-values < 0.05 were statistically significant (bold) regarding a 95% confidence interval (CI).

**Table 3:** Outcome measurements comparisons at baseline among EMS, WB-EMS, and control groups.

Baseline Outcome Measurements	EMS (*n* = 40)	WB-EMS (*n* = 40)	Control (*n* = 40)	*p*-Value
HR (bpm)	76.00 ± 18.25 (51–118)	71.00 ± 18.25 (51–100)	79.00 ± 16.25 (54–116)	0.249 †
SBP (mmHg)	12.30 ± 1.60 (10.30–14.60)	12.40 ± 1.88 (10.50–14.25)	12.30 ± 1.75 (9.10–14.50)	0.236 †
DBP (mmHg)	7.15 ± 1.00 (5.20–8.60)	7.35 ± 1.07 (6.00–9.90)	7.10 ± 1.20 (6.00–9.40)	0.351 †
Temperature (°C)	36.65 ± 0.30 (36.40–36.90)	36.60 ± 0.40 (35.70–36.90)	36.60 ± 0.18 (34.60–36.80)	0.050 †
IRD change (cm)	0.09 ± 0.23 (−0.20–0.61)	0.08 ± 0.11 (−0.19–1.03)	0.09 ± 0.09 (−0.12–0.38)	0.715 †
Right TrA thickness change (cm)	0.05 ± 0.13 (−0.06–0.29)	0.04 ± 0.04 (−0.07–0.17)	0.02 ± 0.07 (−0.09–0.36)	0.108 †
Left TrA thickness change (cm)	0.04 ± 0.10 (−0.07–0.31)	0.02 ± 0.09 (−0.09–0.23)	0.04 ± 0.08 (−0.10–0.42)	0.191 †
Right IO thickness change (cm)	0.05 ± 0.16 (−0.14–0.37)	0.06 ± 0.13 (−0.20–0.28)	0.02 ± 0.09 (−0.14–0.37)	0.270 †
Left IO thickness change (cm)	0.03 ± 0.11 (−0.16–0.30)	0.03 ± 0.10 (−0.17–0.27)	0.04 ± 0.12 (−0.13–0.43)	0.594 †
Right EO thickness change (cm)	−0.03 ± 0.10 (−0.25–0.08)	−0.03 ± 0.10 (−0.25–0.21)	−0.01 ± 0.09 (−0.22–0.14)	0.294 †
Left EO thickness change (cm)	−0.03 ± 0.11 (−0.26–0.14)	−0.02 ± 0.09 (−0.19–0.13)	−0.01 ± 0.08 (−0.15–0.12)	0.069 †
Right RA thickness change (cm)	0.03 ± 0.10 (−0.10–0.25)	0.03 ± 0.06 (−0.05–0.32)	0.03 ± 0.06 (−0.09–0.20)	0.977 †
Left RA thickness change (cm)	0.03 ± 0.09 (−0.08–0.20)	0.04 ± 0.07 (−0.06–0.30)	0.05 ± 0.07 (−0.25–0.34)	0.672 †

Abbreviations: bpm, beats per minute; DBP, diastolic blood pressure; EMS, electrical muscle stimulation; EO, external oblique; HR, heart rate; IO, internal oblique; IRD, inter-rectus distance; RA, rectus anterior; SBP, systolic blood pressure; TrA, transversus abdominis; WB-EMS, whole body electrical muscle stimulation; °C, centigrade degrees. † Median ± interquartile range, and range (minimum–maximum) were used and compared by the Kruskal–Wallis test. *p*-values < 0.05 were statistically significant (bold) regarding a 95% confidence interval (CI).

**Table 4:** Comparisons of the outcome measurement differences among the EMS, WB-EMS, and control groups after interventions.

Outcome Measurements	EMS (*n* = 40)			WB−EMS (*n* = 40)			Control (*n* = 40)			*p*-Value
	Pre	Post	Difference	Pre	Post	Difference	Pre	Post	Difference	
HR (bpm)	78.40 ± 15.49 (51–118)	85.00 ± 22.28 (53–137)	6.60 ± 12.42 (−22–31)	73.47 ± 13.16 (51–100)	105.12 ± 18.12 (59–137)	31.65 ± 16.22 (1–71)	74.10 ± 15.68 (54–116)	100.62 ± 20.81 (59–137)	26.52 ± 16.22 (−4–75)	**<0.001** *
SBP (mmHg)	12.30 ± 1.60 (10.30–14.60)	11.95 ± 2.13 (9.90–14.90)	0.00 ± 0.88 (−3.20–2.90)	12.40 ± 1.88 (10.50–14.25)	12.10 ± 1.30 (9.70–14.70)	−0.30 ± 1.67 (−3.70–2.00)	12.30 ± 1.75 (9.10–14.50)	11.70 ± 1.45 (9.70–14.10)	−0.20 ± 1.30 (−3.40–5.00)	0.665 †
DBP (mmHg)	7.27 ± 0.73 (5.20–8.60)	7.12 ± 0.88 (5.40–9.50)	−0.15 ± 0.94 (−1.90–2.40)	7.53 ± 7.85 (6.00–9.90)	7.60 ± 0.90 (5.50–9.70)	0.06 ± 0.84 (−1.50–2.00)	8.66 ± 9.00 (6.00–9.40)	8.52 ± 8.72 (5.10–9.20)	−0.13 ± 0.78 (−2.00–2.30)	0.439 *
Temperature (°C)	36.65 ± 0.30 (36.40–36.90)	36.55 ± 0.30 (36.80–37.20)	−0.10 ± 0.40 (−1.60–0.70)	36.60 ± 0.40 (35.70–36.90)	36.50 ± 0.38 (35.00–37.90)	−0.10 ± 0.48 (−1.30–1.10)	36.60 ± 0.18 (34.60–36.80)	36.50 ± 0.48 (34.60–36.90)	−0.10 ± 0.50 (−2.00–2.00)	0.755 †
IRD change (cm)	0.09 ± 0.23 (−0.20–0.61)	0.03 ± 0.23 (−0.35–0.45)	−0.07 ± 0.18 (−0.58–0.22)	0.08 ± 0.11 (−0.19–1.03)	−0.01 ± 0.15 (−0.22–0.33)	−0.07 ± 0.18 (−1.09–0.18)	0.09 ± 0.09 (−0.12–0.38)	0.03 ± 0.08 (−0.09–0.15)	−0.09 ± 0.18 (−0.46–0.22)	0.597 †
Right TrA thickness change (cm)	0.05 ± 0.13 (−0.06–0.29)	0.04 ± 0.11 (−0.06–0.28)	0.00 ± 0.07 (−0.19–0.14)	0.04 ± 0.04 (−0.07–0.17)	0.03 ± 0.08 (−0.09–0.15)	0.00 ± 0.08 (−0.14–0.15)	0.02 ± 0.07 (−0.09–0.36)	0.01 ± 0.07 (−0.05–0.27)	0.00 ± 0.07 (−0.14–0.26)	0.952 †
Left TrA thickness change (cm)	0.04 ± 0.10 (−0.07–0.31)	0.04 ± 0.08 (−0.03–0.19)	0.00 ± 0.08 (−0.19–0.17)	0.02 ± 0.09 (−0.09–0.23)	0.02 ± 0.06 (−0.08–0.16)	0.01 ± 0.08 (−0.15–0.21)	0.04 ± 0.08 (−0.10–0.42)	0.03 ± 0.08 (−0.09–0.49)	−0.01 ± 0.10 (−0.35–0.18)	0.644 †
Right IO thickness change (cm)	0.05 ± 0.16 (−0.14–0.37)	0.05 ± 0.10 (−0.12–0.39)	0.00 ± 0.11 (−0.37–0.29)	0.06 ± 0.13 (−0.20–0.28)	0.05 ± 0.13 (−0.04–0.41)	0.01 ± 0.09 (−0.29–0.18)	0.02 ± 0.09 (−0.14–0.37)	0.04 ± 0.10 (−0.15–0.44)	0.01 ± 0.13 (−0.26–0.26)	0.819 †
Left IO thickness change (cm)	0.03 ± 0.11 (−0.16–0.30)	0.03 ± 0.12 (−0.13–0.20)	0.01 ± 0.11 (−0.43–0.20)	0.03 ± 0.10 (−0.17–0.27)	0.03 ± 0.15 (−0.15–0.35)	0.02 ± 0.13 (−0.27–0.23)	0.04 ± 0.12 (−0.13–0.43)	0.06 ± 0.08 (−0.10–0.34)	0.02 ± 0.10 (−0.29–0.27)	0.780 †
Right EO thickness change (cm)	−0.03 ± 0.10 (−0.25–0.08)	−0.01 ± 0.10 (−0.18–0.16)	0.01 ± 0.10 (−0.12–0.26)	−0.03 ± 0.10 (−0.25–0.21)	−0.02 ± 0.11 (−0.21–0.10)	0.00 ± 0.12 (−0.40–0.28)	−0.01 ± 0.09 (−0.22–0.14)	−0.01 ± 0.07 (−0.16–0.13)	0.01 ± 0.08 (−0.14–0.21)	0.816 †
Left EO thickness change (cm)	−0.04 ± 0.08 (−0.26–0.14)	−0.03 ± 0.07 (−0.20–0.12)	0.01 ± 0.07 (−0.24–0.20)	−0.01 ± 0.07 (−0.19–0.13)	0.00 ± 0.08 (−0.18–0.18)	0.01 ± 0.09 (−0.20–0.24)	−0.01 ± 0.06 (−0.15–0.12)	−0.00 ± 0.07 (−0.16–0.20)	0.01 ± 0.07 (−0.17–0.19)	0.792 *
Right RA thickness change (cm)	0.03 ± 0.10 (−0.10–0.25)	0.02 ± 0.10 (−0.06–0.13)	−0.01 ± 0.08 (−0.17–0.12)	0.03 ± 0.06 (−0.05–0.32)	0.03 ± 0.07 (−0.08–0.21)	0.00 ± 0.06 (−0.15–0.10)	0.03 ± 0.06 (−0.09–0.20)	0.05 ± 0.06 (−0.06–0.16)	0.01 ± 0.05 (−0.08–0.23)	0.264 †
Left RA thickness change (cm)	0.03 ± 0.09 (−0.08–0.20)	0.03 ± 0.10 (−0.12–0.12)	−0.01 ± 0.06 (−0.19–0.14)	0.04 ± 0.07 (−0.06–0.30)	0.04 ± 0.09 (−0.04–0.22)	0.00 ± 0.07 (−0.15–0.15)	0.05 ± 0.07 (−0.25–0.34)	0.05 ± 0.06 (−0.07–0.13)	−0.01 ± 0.08 (−0.26–0.32)	0.381 †

Abbreviations: bpm, beats per minute; DBP, diastolic blood pressure; EMS, electrical muscle stimulation; EO, external oblique; HR, heart rate; IO, internal oblique; IRD, inter-rectus distance; RA, rectus anterior; SBP, systolic blood pressure; TrA, transversus abdominis; WB-EMS, whole body electrical muscle stimulation; °C, centigrade degrees. * Mean ± standard deviation, and range (minimum–maximum) were used and compared by the one-way analysis of variance (ANOVA). † Median ± interquartile range, and range (minimum–maximum) were used and compared by the Kruskal–Wallis test. *p*-values < 0.05 were statistically significant (bold) regarding a 95% confidence interval (CI).

## Data Availability

The data presented in this study are available on request from the corresponding author.
